# Peritoneal Inclusion Cyst in a Young Patient With a Long History of Abdominal Surgeries: A Case Report

**DOI:** 10.7759/cureus.35230

**Published:** 2023-02-20

**Authors:** Jawahir O AlTamimi, Esraa A Alzahrani, Abdullah Fallatah, Lujain A Alhakami, Bayan E Bokhari

**Affiliations:** 1 College of Medicine, King Saudi bin Abdulaziz for Health Sciences, Jeddah, SAU; 2 General Surgery, King Abdulaziz Medical City, Jeddah, SAU; 3 College of Medicine, King Saud Bin Abdulaziz University for Health Sciences, Jeddah, SAU; 4 General/Colorectal Surgery, King Abdulaziz Medical City, Jeddah, SAU

**Keywords:** chronic urinary incontinence, bladder exstrophy, laparoscopic surgery, lower abdominal surgery, peritoneal inclusion cyst

## Abstract

Peritoneal inclusion cysts (PICs) are reactive, fluid-filled lesions of the peritoneal lining, usually affecting women of reproductive age and with previous abdominal surgeries. Paraovarian cysts, hydrosalpinx, and low-grade cystic mesothelioma are usually considered in the differential diagnosis of PICs. In this case report, we present an 18-year-old female with a known case of bladder exstrophy and chronic urinary incontinence and a previous history of surgical bladder repair. She presented to the emergency department (ED) with urinary incontinence and lower abdominal pain. A computed tomography was ordered for her to rule out hydronephrosis, and incidentally, ovarian cysts were discovered that were then bilaterally excised via laparotomy. Our case report emphasizes the significance of considering such a diagnosis when coming across patients whose risk factors and symptoms match the diagnosis.

## Introduction

Peritoneal inclusion cysts (PICs) are reactive, fluid-filled lesions of the peritoneal lining [1]. Women of reproductive age are usually affected by the disease, especially if they have a previous history of abdominal surgery, inflammation, or infection. Patients usually present with lower abdominal pain, pelvic fullness, and sometimes a palpable mass [2]. However, 10% of PICs are diagnosed incidentally in asymptomatic patients during imaging or surgery. PICs usually appear as multilocular cystic lesions that are filled with fluid, adjacent to pelvic organs, and adherent to ovaries [3]. Paraovarian cysts, hydrosalpinx, and low-grade cystic mesothelioma are usually considered in the differential diagnosis of PICs. Fortunately, PICs have no malignant potential; therefore, a conservative approach can be considered as an alternative to surgery. Image-guided aspiration combined with oral contraceptives is the most effective method. However, fluid re-accumulation will eventually occur. Thus, many patients choose surgical intervention [1]. Here, we present a case of bilateral PICs in an 18-year-old female patient with a history of bladder repair.

## Case presentation

An 18-year-old female with a known case of bladder exstrophy and chronic urinary incontinence underwent bladder repair and bladder neck reconstruction in 2016 and 2018, respectively. Despite these multiple repairs, the patient still suffers from urinary incontinence and presented to the emergency department (ED) complaining of urinary retention, lower abdominal pain radiating to the flanks, and dysuria. The patient was on clean intermittent catheterization (CIC), which she stopped six months ago because the patient was able to void freely. During the family history revision, the patient denied any genetic disorders in the family. She was not on any medications.

Upon physical examination, the abdomen had multiple scars from previous surgeries; the abdomen was distended and soft with mild tenderness in the suprapubic area. There was no rigidity or guarding.

Her urinalysis showed positive nitrates, traces of protein, moderate red blood cells (RBCs) and white blood cells (WBCs), and moderate blood; therefore, she was admitted with a case of urinary tract infection and was started on antibiotics intravenously for two weeks.

**Figure 1:**
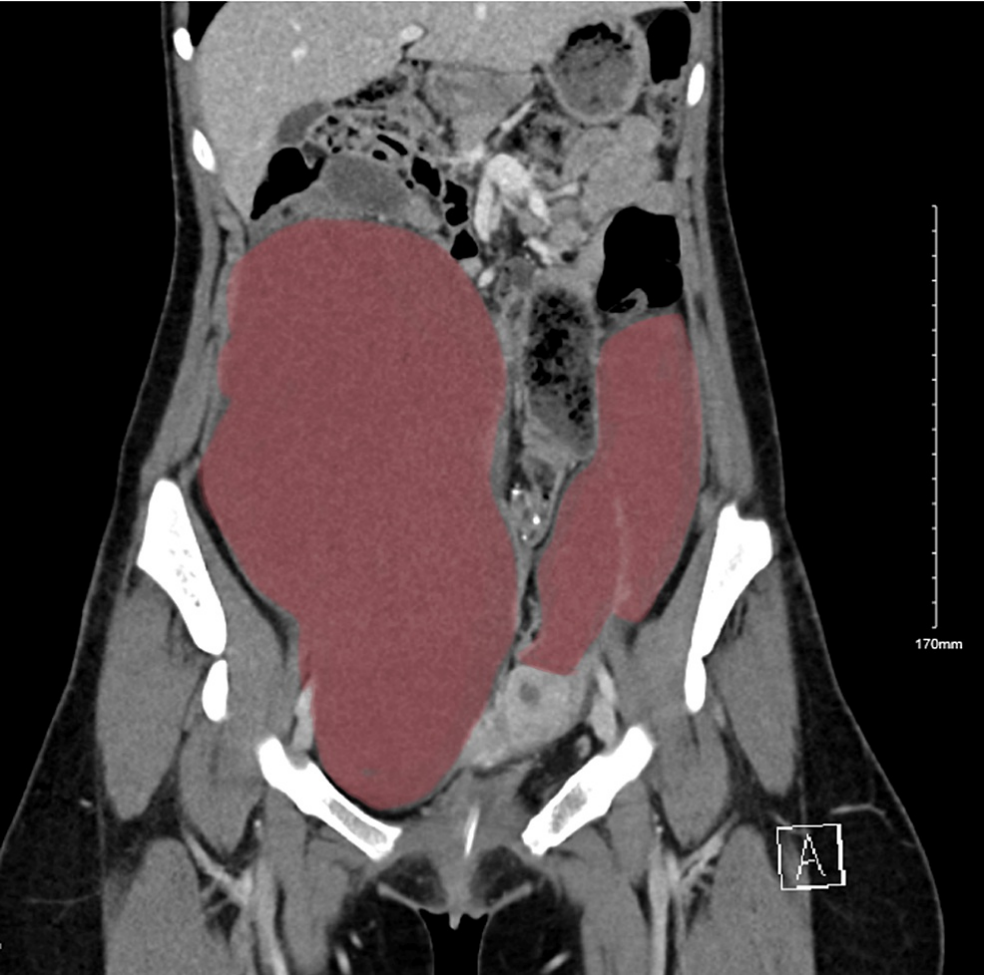
CT scan of the abdomen and pelvis of the bilateral peritoneal cysts in the coronal view.

**Figure 2:**
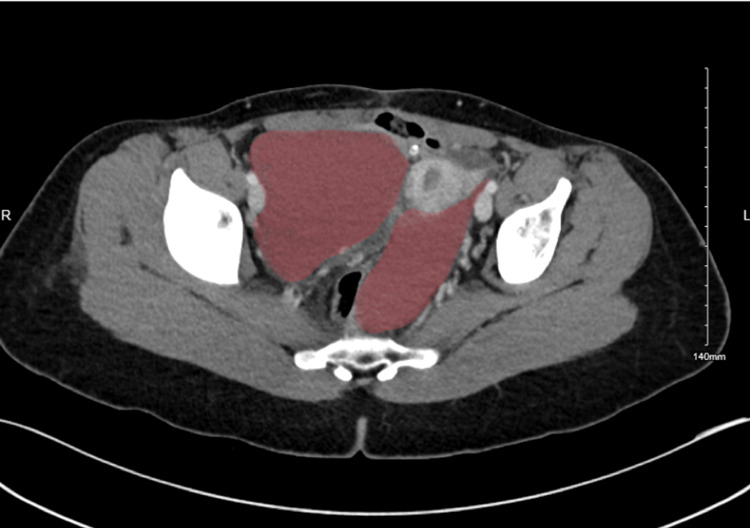
CT scan of the abdomen and pelvis of the bilateral peritoneal cysts in the transverse view.

Abdominopelvic computed tomography (CT) was ordered to rule out hydronephrosis. It illustrated smooth, diffuse bilateral urothelial enhancements, most likely related to inflammation. In addition, as an incidental finding, it showed bilateral elongated abdominopelvic cystic masses along the bilateral adnexal vessels and ovaries. The right and left masses measured 5.8 cm × 12 cm × 2.4 cm and 9 cm × 6 cm × 15 cm, respectively (Figures 1-2). Further evaluation via pelvic magnetic resonance imaging (MRI) was needed to determine the origin of the cyst, which demonstrated non-complicated cystic lesions bilaterally, most likely to be peritoneal inclusion cysts, and also showed evidence of uterine duplication anomalies.

**Figure 3:**
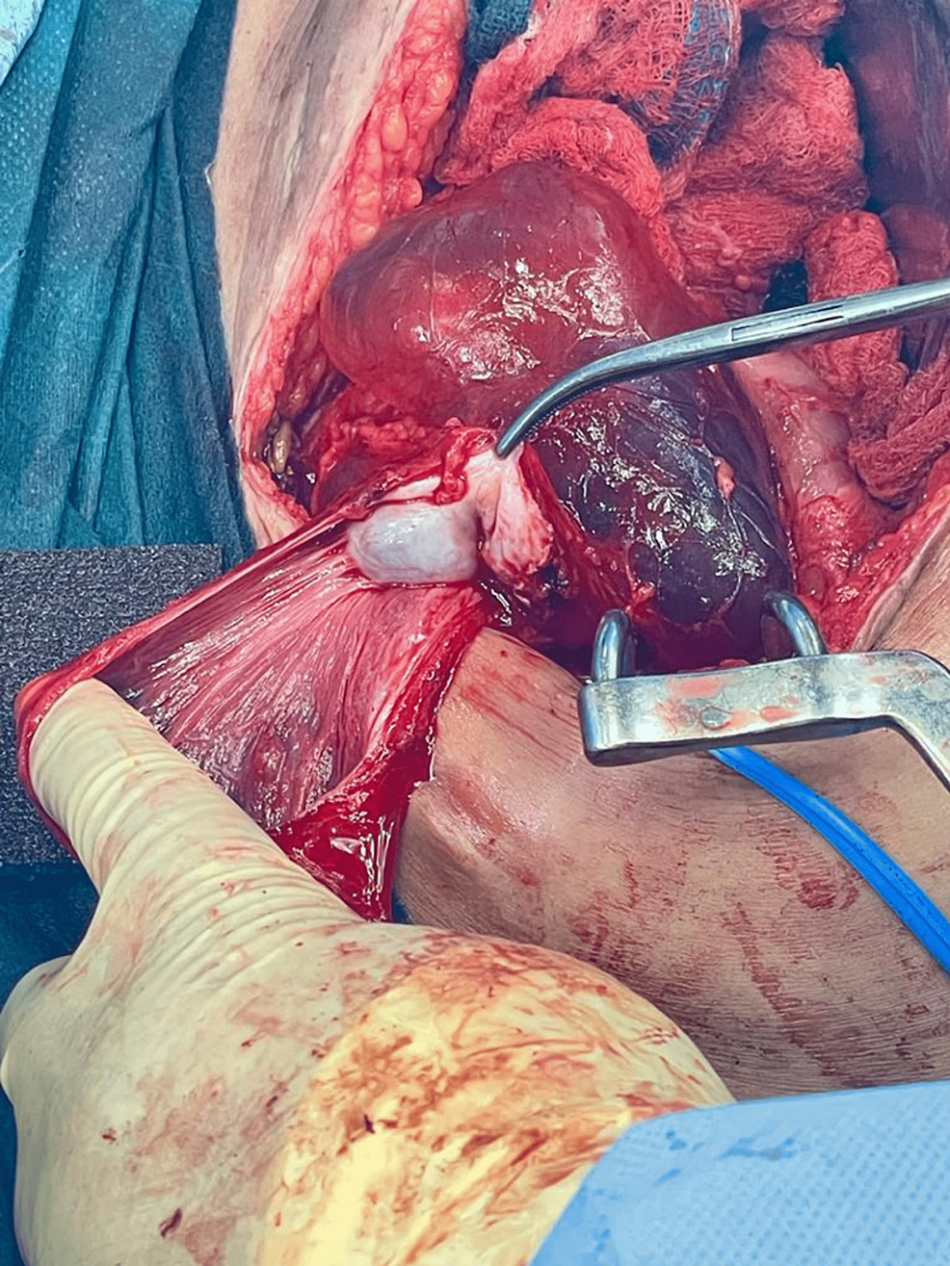
Left open cyst showing normal ovary

**Figure 4:**
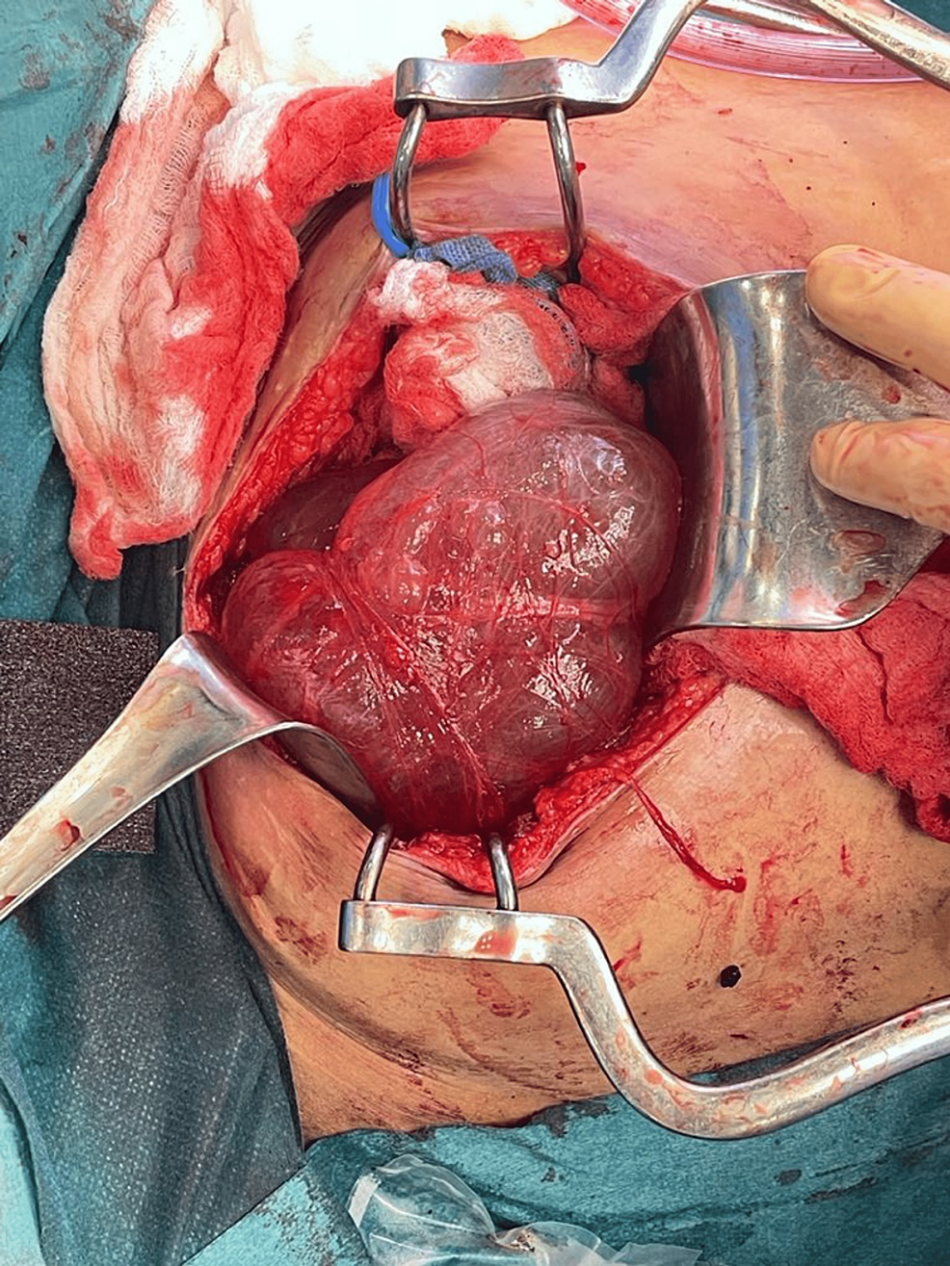
Left peritoneal cyst

**Figure 5:**
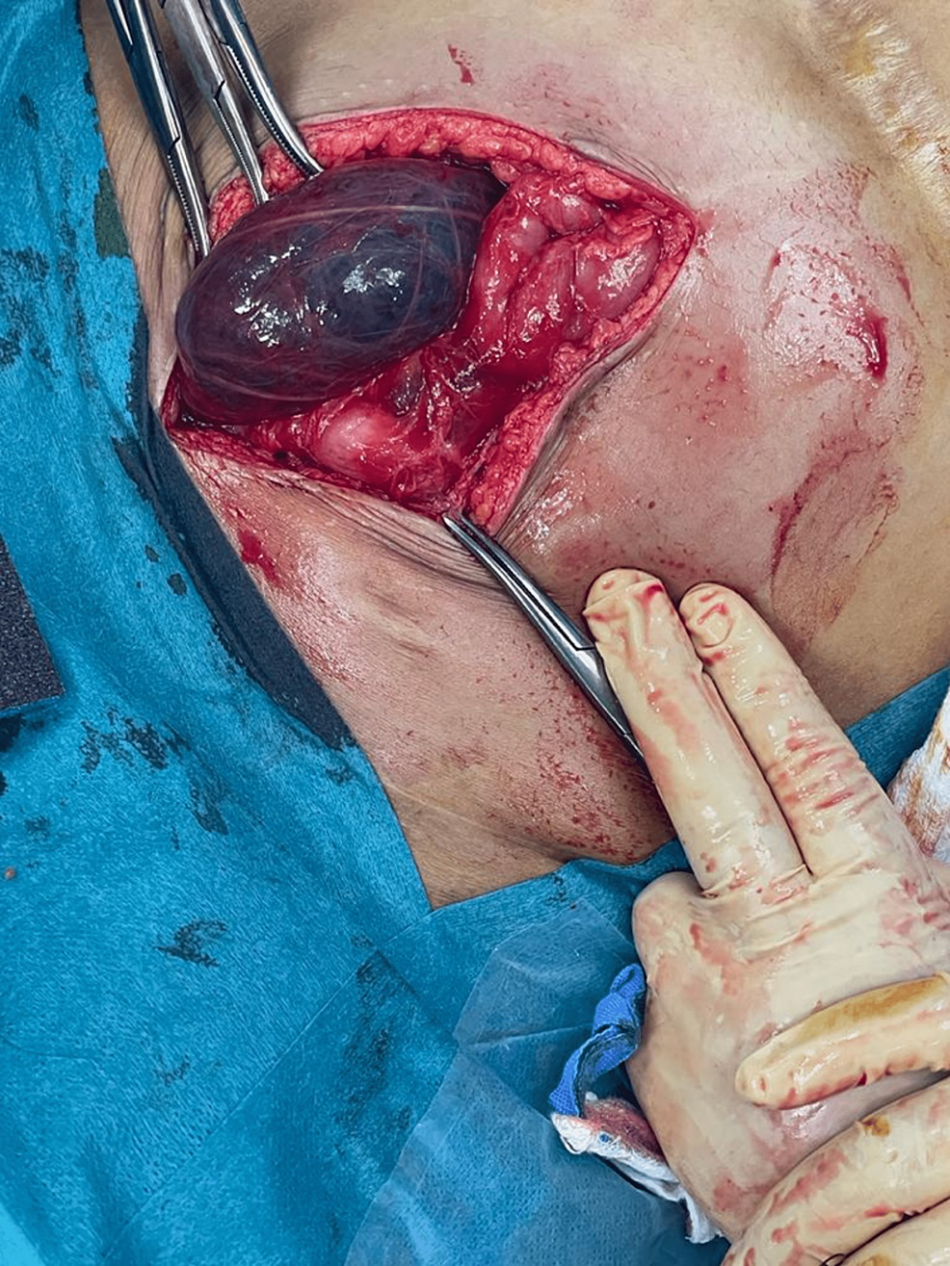
Right peritoneal cyst

The patient underwent cyst drainage under interventional radiology. They drained brown fluid, and pathology showed no evidence of malignant cells. The patient’s pain level decreased, and she was discharged home. After two weeks, she had the same complaint, and her abdominal pain became more severe. But there were no signs of infection. A CT of the abdomen and pelvis was repeated, and it showed fluid reaccumulating within the cyst. After discussing with the patient that the risk of recurrence of PIC is still high despite surgical resection, the decision to do a laparotomy with bilateral excision of the peritoneal inclusion cyst was made (Figures 3-5). Intraoperatively, there were severe adhesions, and the sigmoid colon was injured by a less than 1 cm laceration. In addition, colocutaneous fistula and pelvic collections developed post-operatively. The fistula was managed conservatively for two weeks with complete resolution, and the fluid collections were drained by interventional radiology.

The pathology report demonstrated a peritoneal (multilocular) inclusion cyst bilaterally.

## Discussion

Although the etiology behind peritoneal inclusion cysts is still unclear, they are thought to be the result of benign inflammatory proliferation due to various risk factors like prior abdominopelvic surgeries, gastrointestinal inflammation, or pelvic inflammation [4]. In regard to our case’s history, the most likely hypothesis behind her PIC development is peritoneal disruption via prior surgery, leading to impaired fluid absorption and ultimately arising as a cyst [5].

Peritoneal inclusion cysts are often found at the bottom of the differential diagnosis list when addressing the associated symptoms in patients with prior abdominopelvic surgeries or gastrointestinal or pelvic inflammation. In our case, urinary tract infection and hydronephrosis were the main differentials given our patient’s history of bladder repair surgery. It is stated in the literature that 10% of cases are incidentally diagnosed, and our case is no exception [2]. A CT initially ordered to rule out hydronephrosis revealed the bilateral cystic masses.

Various treatment options are offered to treat peritoneal inclusion cysts. Such different modalities of treatment include but are not limited to observation, hormonal management, image-guided aspiration, image-guided sclerotherapy, potassium-titanyl-phosphate laser ablation, and surgical excision [4]. Elective surgeries, such as in our case, are usually the most common treatment option due to persistent symptoms, definitive management, and the patient’s preference. Both laparoscopic and laparotomic approaches are valid options for PCI excision; however, laparoscopic surgery is the preferred method because it offers less blood loss and a shorter hospital stay, but the rate of recurrence was the same for both techniques [6]. Despite their benign features and low malignant potential, peritoneal inclusion cysts have a high rate of recurrence irrespective of procedures, around 30-50% [4].

The multitude of presentations and subsequent treatment of peritoneal inclusion cysts is evident in the literature. Singh et al. reported a multiparous woman with a history of bilateral tubal ligation who presented with gradual lower abdominal pain [7]. Her CT showed fibroids and numerous, undefined cysts that mimicked a large ovarian tumor. Ultimately, she underwent staging laparotomy only to find healthy ovaries and multiple peritoneal inclusion cysts, confirmed by histopathology [7]. In a case report by Tamai et al., a middle-aged woman also presented with progressive lower abdominal pain but had a history of left ovarian cystectomy [8]. On ultrasonography, an intramural myoma was appreciated, but a subsequent MRI was ordered to better visualize the left ovary, revealing peritoneal inclusion cysts [8]. Aspiration and drainage were both diagnostic and therapeutic [8]. As stated in the reports, the common denominator amongst them and our case is symptomatic presentation, such as increasing lower abdominal pain as well as having a prior history of abdominopelvic surgeries. However, an individualized course of treatment was tailored to each case. Similar to our case, initial symptomatic relief was obtained through cyst drainage in Tamai et al. However, subsequent treatment was different, as our patient was treated by inclusion cyst excision, while Tamai et al. resorted to conservative hormonal therapy [8].

## Conclusions

Although the occurrence of peritoneal inclusion cysts is relatively low, the rising evidence alongside our case reports emphasizes the significance of considering such a diagnosis in patients whose risk factors and symptoms match the diagnosis. Although the rate of mortality is low, prompt diagnosis followed by definitive management can aid in decreasing the high morbidity rate associated with peritoneal inclusion cysts. Management is best tailored to each individualized case as the pathogenesis is still unclear and the recurrence rate is high irrespective of the procedure. The literature still needs continuous data to effectively establish guidelines for approaching peritoneal inclusion cysts.
